# Development, validation and evaluation of an online medication review tool (MedReview)

**DOI:** 10.1371/journal.pone.0269322

**Published:** 2022-06-03

**Authors:** Kaeshaelya Thiruchelvam, Syed Shahzad Hasan, Alex Pudmenzky, Wong Pei Se, Therese Kairuz

**Affiliations:** 1 School of Pharmacy, International Medical University, Kuala Lumpur, Malaysia; 2 School of Biomedical Sciences and Pharmacy, University of Newcastle, Newcastle, New South Wales, Australia; 3 Department of Pharmacy, University of Huddersfield, Huddersfield, United Kingdom; 4 School of Business, University of Queensland, Brisbane, Australia; UCSI University, MALAYSIA

## Abstract

**Objectives:**

To develop, validate and evaluate a computerized clinical decision support system (MedReview) that aids medication reviewers with pharmacological decision-making.

**Methods:**

This study included three phases; the development phase included computerizing a consolidated medication review algorithm (MedReview), followed by validation and evaluation of MedReview and responding to a web-based survey designed using patient scenarios. Participants had to be ‘fully registered’ with the Malaysian Pharmacy Board and work full-time at a community pharmacy.

**Results:**

MedReview was developed as a web app. It was validated among 100 community pharmacists from May-July 2021 using the Technology Acceptance Model (TAM). There was acceptable content validity and fair inter-rater agreement, and good convergent and discriminant validity. Exploratory factor analysis resulted in five domains to determine the attitude of pharmacists about using MedReview: perceived ease of use, perceived usefulness, intention to use, trust, and personal initiatives and characteristics; the total variance explained by five factors was 76.36%. The survey questionnaire had a high overall reliability value of 0.96. Evaluation of MedReview was based on mean scores of survey items. Of all items included in the survey, the highest mean score (out of 7) was achieved for ‘I could use MedReview if it is meaningful/relevant to my daily tasks’ (5.78 ± 1.10), followed by ‘I could use MedReview if I feel confident that the data returned by MedReview is reliable’ (5.77 ± 1.21), and ‘I could use MedReview if it protects the privacy of its users’ (5.73 ± 1.20).

**Conclusion:**

Community pharmacists generally had a positive attitude towards MedReview. They found that MedReview is trustworthy and they had the intention to use it when conducting medication reviews. The adaptation of the TAM in the survey instrument was reliable and internally valid.

## Introduction

Medication review is ‘a structured evaluation of a patient’s medicines with the aim of optimizing medicines use and improving health outcomes. This entails detecting drug-related problems and recommending interventions’ [1]. The medication review process is usually conducted by pharmacists, and promotes appropriate polypharmacy, aids in identifying possible and true medication-related adverse events, offers an opportunity to promote medication adherence, and reduces the use of potentially inappropriate medications (PIMs) [1]; PIMs are medications whose potential risk of adverse effects outweighs the benefits among older people [2].

Current medication review processes have certain limitations, including lack of a systematic and structured approach which leads to variations in the process; time and funding constraints; lack of integration at residential aged care facilities (RACFs) and lack of data storage to enable quality improvement; and the inability to conduct face-to-face reviews especially due to the coronavirus disease-19 (COVID-19) pandemic [3]. Medication review algorithms and minimization frameworks are used as guides in the medication review process, and aid reviewers in identifying medication-related problems thereby reducing harmful events among the older population [4]. Although many algorithms and minimization frameworks have been developed [4–6], they are not usually tailored to the older population, and lack a scoring scheme to quantify the medication review process; the latter may preclude periodical monitoring of improvements or deterioration.

A consolidated medication review algorithm by Thiruchelvam et al. was peer-reviewed in 2018 [7]. A key difference from other medication review algorithms and frameworks is the incorporation of the PIM component using the Beers Criteria [2], making it relevant to the older population. It includes a scoring scheme that corresponds to components in the algorithm which allow for periodical monitoring of health associated with medication use. Scores are generated in a manner similar to the Medication Appropriateness Index [8]; medication aspects deemed appropriate are scored 0 while inappropriate aspects are scored 1. Medication aspects included in the consolidated algorithm are: indication, effectiveness, costs, therapeutic duplication, identification of PIMs and medications to be used with caution, as well as contraindications, incidence of adverse drug events, appropriateness of dosing regimen, and previous discontinuations [7].

In pharmacy practice, the use of technology was implemented in 2012 [9]. Technology in the form of computerized clinical decision support systems (CDSS) ensures that medication reviews are conducted in a timely and efficient manner. CDSS is a software application that uses databases of patients’ personal and clinical information, producing patient-specific pharmacological recommendations via an embedded algorithm [10]. CDSS has been proven to promote the appropriate and safe use of medications for older people [11]; however, it is important that it is tested for validity and reliability.

In Malaysia, pharmacist-led medication reviews are not an established and remunerated service funded by the Government. Nevertheless, an informal manner of medication reviews may be conducted by pharmacists working in government-owned public health clinics and hospitals where the majority of Malaysian pharmacists are employed [12]. However, it has been reported that the clinical skills of community pharmacists are underutilised [13]. Less than two prescriptions a day are filled at community pharmacies in Malaysia because private general practitioners (GPs) were granted rights under the Poison Act 1952 to dispense medications in their clinics [14]. Studies indicate that GPs seldom conduct medication reviews for patients with repeat prescriptions [15,16]. Healthcare reform to improve the effectiveness, efficiency, equity and consumers’ choice has become a key agenda for policy change in Malaysia [17]. Given that community pharmacists have medication expertise and are easily accessible to the general public, they should be able to positively contribute to the objectives of the reform [12].

Given the track record of CDSS in improving health outcomes in older people, we hypothesized that the consolidated medication review algorithm developed by Thiruchelvam et al. [7], and used in the form of a CDSS, would assist pharmacists during medication reviews. Community pharmacists have the potential to expand their scope of practice to review medications for patients at risk of medication-related problems. As such, it is important to determine their intention and attitude in using a CDSS that incorporates a medication review algorithm. Therefore, the objectives of this study were to develop a CDSS that incorporated the original consolidated medication review algorithm by Thiruchelvam et al., and validate and evaluate the CDSS among community pharmacists in Malaysia.

## Methods

The development of the CDSS ‘MedReview’ was based on the consolidated medication review algorithm [7] and performed by an app developer. The validation and evaluation phases were based on responses to a web-based survey after participants had completed medication reviews using MedReview, for hypothetical patients and using case scenarios.

### Development of MedReview

The following sections describe the development and use of MedReview in our study.

#### Platform independence

The software was developed as a web app as opposed to a native app to avoid users having to download and install the software; a native app is one that is built for a specific platform. Since it was not known whether the target audience used iOS, Android or a Windows mobile platform or desktop computers, the web app provided a platform-independent approach without compromising any functionality, since it did not require any embedded devices including global positioning system (GPS), camera, microphone and accelerometer. Creating a web app allows the software to run within a browser from any device, mobile phone or desktop, which increases user uptake.

#### Graphical user interface

The Graphical User Interface (GUI) was developed using the jQuery mobile v1.4.5 framework [18]. jQuery Mobile is a HTML5-based user interface system designed to make responsive web sites and apps. It is built on jQuery and jQuery UI which are lightweight JavaScript libraries that allow for a flexible and easily themeable design.

#### Back-end software

The back-end software was developed in PHP v7.3 with MySQL v8.0. The MySQL database contained the text for the user instructions and resources that the user was able to display on the GUI, the users’ credentials (passwords were stored salted and hashed) and the yes/no questions used during the interview. Each question was given a number, and the question numbers to branch to if the answer given was either yes or no. Special code was implemented to prohibit any infinite loops being created accidentally (by directing a question back to a previously asked question).

#### Functionality

**Fig 1:**
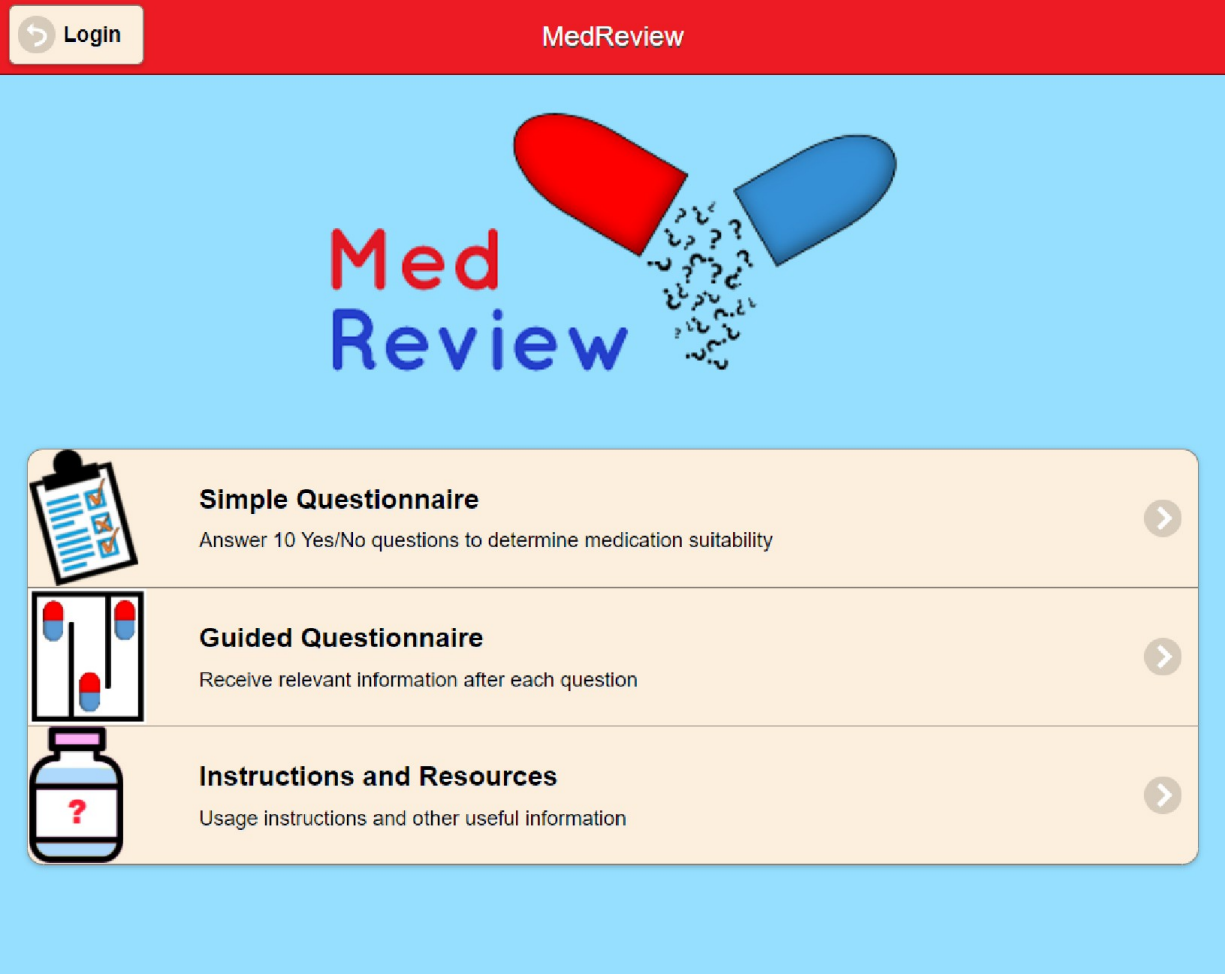
Screenshot of the home page of MedReview.

Fig 1 depicts the homepage of MedReview. Users registered at https://smartmedreview.com/ using their email address only and received a password via return email; functions to change an assigned password and to recover a forgotten password were also implemented. Since the app should not store personal information for privacy reasons, no additional security requirements such as password expiry or forced password change were implemented. The reason why registration via an email address was required was to email the calculated medication scores back to the user. A list of all users was generated for evaluation purposes in this study.

After entering their credentials, the user was presented with an option to execute either the Simple Questionnaire or the Guided Questionnaire, or to read the instructions and other resources. Both questionnaires began by requesting the ‘name’ of the patient whose medication was to be evaluated. No input validation was performed on these fields and the user could enter pseudonyms, for privacy reasons.

The Simple Questionnaire contained 10 yes/no questions for each medication included in the review, which were presented on a single screen; the yes/no answers contribute to a score [7]. Although the Simple Questionnaire can be done quickly, it does not provide a stepwise guide with inbuilt suggestions/recommendations. The Guided Questionnaire, on the other hand, used a stepwise approach with yes/no questions specifically designed to facilitate a comprehensive medication review. Each subsequent question depended on the answer to the previous question. Scores were generated in a manner similar to the Simple Questionnaire.

Once completed, the resulting score (range 0–10) for each medication was displayed. A questionnaire could be completed for as many medications as required for each patient. The score for each medication (referred to as the Medicine Score) was calculated, where 0 represented extremely appropriate and 10 represented extremely inappropriate. MedReview also calculated an average final score (Medication Average Score), which represented the sum of scores for individual medicines. For example, a high Medicine Score indicated the need to address medication appropriateness.

The section ‘Instructions and Resources’, provided a detailed overview of instructions on how to use the questionnaires, and resources that may be helpful to pharmacists, including links to UpToDate^®^ and Lexicomp^®^.

### Validation of MedReview

#### Study population and data collection

Data were collected from a cross-sectional sample of community pharmacists in Malaysia. Participants were included if they were ‘fully-registered’ pharmacists with the Malaysian Pharmacy Board, worked full-time at a community pharmacy and provided informed consent to participate. Participants were identified via convenience sampling (personal contacts, social media, owners of pharmacies); those interested in participating were briefed via text messaging and e-mail. Data collection was completed in two months (May-July 2021).

Participants were provided with a study information sheet as per ethical requirements, and a user guide which included study instructions and a hypothetical case; participants were required to use MedReview to perform a medication review for this ‘case’. The hypothetical case was a ‘prescription’ received at a (hypothetical) pharmacy. For the purposes of this study, participants were required to register to use MedReview so that the recommendations generated by MedReview could be emailed to them. The research team received a copy of these results for analysis. After submitting their cases, participants received a web link to the survey about their experience using MedReview.

#### The survey instrument

Various models have been developed to test user intention and attitude to adopt new technologies, including the Technology Acceptance Model (TAM) [19]. The TAM was developed by Davis et al. (1989) to determine user acceptance of technology within the business and information technology sector. With regards to TAM, information technology is ‘more adopted’ if it is useful, and it is ‘more accepted’ if it is easy to use [19]. Most researchers who have adopted technology in healthcare and have used the TAM generally concur with these constructs [20–22].

The survey in this study was provided as a Google Survey link and comprised two sections: the first included demographic information about the participants, while the second contained items (questions) for the validation and evaluation of MedReview. The items were adapted from the TAM by Davis et al. (1989) (perceived usefulness and perceived ease of use) [19], and Gao et al. (2011) [21]. The reliability (Cronbach’s alpha coefficient) of both constructs (usefulness and ease of use) in Davis et al. were 0.98 and 0.94 [19], respectively, and the reliability of other constructs as per Gao et al. was above 0.70 [21]. All items in our survey had a 7-point Likert scale where participants indicated their level of agreement with 1 being strongly disagree and 7 being strongly agree.

Content and face validation were conducted among four pharmacists with experience/expertise in medication use and medication reviews. Content validation ensured that the survey instrument was relevant and representative of the targeted constructs [23], while face validation ensured that the items measured what they intended to measure [24]. The content validation process comprised two parts, i.e. performing a medication review using MedReview, and indicating the usefulness (useful/not useful) of each item via the survey. Pharmacists were grouped in pairs, and their opinions on the usefulness of each item (between pairs) were quantified using the calculated level of inter-rater agreement. The overall agreement of usefulness was determined based on the following formula: (Agreement of usefulness of first rater pair + agreement of usefulness of second rater pair)/2. Items were excluded only if both pairs agreed with exclusion of items, i.e. overall agreement score of 0. ‘Expert pharmacists’ also provided feedback for face validation, and feedback was collated for improvements to the survey instrument, study information sheet and user guide. Construct validity and internal consistency were also determined.

#### Statistical analysis

The Statistical Package for Social Sciences (SPSS^®^ version 27) with a significance level of 0.05 was used for data analyses. Data are descriptively presented as frequencies, percentages, mean/median and standard deviation/interquartile range. Score distributions across survey constructs were examined.

Construct validation was conducted as the survey instrument had not been previously used in Malaysia. Factorial validity was examined using exploratory factor analysis (EFA) with a principal component analysis (PCA) method. PCA examines the relationship between individual item variances as well as total variances shared between items [25]. The hypothesized model included 30 items of the TAM, and its extension/adaptation [21]. A sample-based EFA method was used, i.e. Kaiser’s alpha factoring, so that the developed instrument may be used for other data sets in future. Factors were rotated using varimax orthogonal rotation, which improves the solution compared to unrotated ones and allows factors to be independent of one another. In the analysis, items with factor loadings >0.40 were considered stable [26]. The Kaiser rule (Kaiser-Meyer-Olkin test (KMO)) and Eigenvalue of >1 were used to measure sample adequacy. However, because the use of >1 as a cut-off value may introduce potential bias [27], the scree plot was inspected as a superior factor selection method in order to determine the adequate number of factors to retain for the rotation. Bartlett’s test of sphericity measured significant correlations between variables.

Finally, criterion validation was examined. Convergent (if items loaded highly on their factors) and discriminant validity (presence of cross-loadings and/or strong correlations between factors, i.e. factor loadings >0.75) were assessed [25].

Internal consistency/reliability of the survey instrument was determined using Cronbach’s alpha (α), where the alpha coefficient determines the extent to which multiple indicators belong together for a latent variable [28]. A commonly acceptable threshold for the reliability is ≥0.70, although values below 0.70 are deemed acceptable [29], and corrected item-total correlation values of individual items should be >0.400 to indicate good correlation [20].

### Evaluation of MedReview

MedReview was evaluated quantitatively and qualitatively in two stages based on the intention and attitude of community pharmacists using MedReview. Stage one was based on open-ended additional feedback from ‘expert pharmacists’ during content and face validation. Following the feedback, changes were made to MedReview prior to data collection. Stage two of the evaluation was conducted among all participants based on descriptive analyses of items (questions) which were available in the second section of the Google Survey link. Participants could also provide free text feedback regarding overall use of MedReview.

### Ethics approval

Our study complies with the standards of the Helsinki declaration pertaining to the investigation of human subjects. The International Medical University Joint Committee on Research and Ethics (Project ID: PHMS I/2021(02)) provided ethical approval for the study. Written informed consent was obtained from each participant prior to data collection. Personal data of all participants were stored in a password-protected file which was accessible only to the study investigators. Personal data will not be disclosed, and study results are reported as de-identified data.

## Results

### Socio-demographic characteristics

Approximately 124 community pharmacists from privately-owned pharmacies were invited to participate and of these, 100 pharmacists participated in the study (average response rate of 80%). The majority of participants were females (n = 65; 65%) and aged between 26 and 35 years old (n = 68; 68%). Most participants (n = 46; 46%) had practiced pharmacy for a mean duration of median of <5 years, while only 7 (7%) had practiced for >20 years. The mean duration of practicing as community pharmacists was 6.33 ± 5.92 years. Demographic characteristics are summarised in S1 Table. Participants were recruited from around Malaysia; the majority resided in Selangor (n = 27; 27%), followed by Johor (n = 19; 19%) and Kuala Lumpur (n = 17; 17%), Malaysia’s capital city.

### Validation of MedReview

The survey showed acceptable content validity, i.e. fair inter-rater agreement. All 30 items in the survey instrument were deemed ‘useful’ during the face and content validation phases (see S2 Table). No questions (items) in the survey had missing responses.

Results of the EFA and factor analysis are depicted in Table 1. The KMO test of sampling adequacy resulted in an overall index of 0.91, indicating that the sample was adequate for factor analysis. Bartlett’s test of sphericity revealed that the inter-correlation matrix was factorable (chi-square (435) = 2970.8, p<0.001). Inspection of the scree plot (S1 Fig), and determining an “elbow” point after which inclusion of additional factors will not result in substantial gains in the “variance explained”, produced a five factor solution (five constructs) for the items in the survey (with Eigenvalues >1); the total variance explained by five factors was 76.36%. The factors were named with regards to items having the highest loadings. According to the results, 20.94% of the variance was explained by the first factor, labelled ‘perceived ease of use’. The variance explained by other factors were: ‘perceived usefulness’ (19.40%), ‘intention to use’ (14.95%), ‘trust’ (14.82%), and ‘personal initiatives and characteristics’ (6.34%). In terms of criterion validation, all 30 items had factor loadings >0.40 with some cross-loading between factors.

**Table 1:** Summary of factor analysis, item-total score correlations, and reliability of the survey (construct validation).

Items	Rotated factors coefficients				
	PE	PU	IU	TR	PIC
Learning to operate MedReview would be easy for me.	**0.802**	0.205	0.158	-0.009	-0.044
I would find it easy to get MedReview to do what I want it to do.	**0.690**	0.443	0.284	0.109	0.093
My interaction with MedReview would be clear and understandable.	**0.700**	0.383	0.182	0.096	0.263
I would find MedReview to be flexible to interact with.	**0.556**	0.387	0.131	0.037	0.527
It would be easy for me to become skilful at using MedReview.	**0.847**	0.155	0.146	0.151	0.032
I would find MedReview easy to use (user-friendly).	**0.855**	0.160	0.167	0.135	0.048
I would find the user interface of MedReview clear and intuitive.	**0.865**	0.095	0.139	0.086	0.106
I am capable of using MedReview.	**0.678**	0.234	0.165	0.159	0.193
I have fun using MedReview.	**0.547**	0.355	0.499	0.144	0.268
I could use MedReview if I am out of home or at my workplace.	**0.534**	0.125	0.308	0.503	0.084
Using MedReview in my job would enable me to accomplish medication reviews more quickly	0.150	**0.853**	0.180	0.138	0.187
Using MedReview would improve my performance in performing a medication review	0.390	**0.753**	0.163	0.171	0.037
Using MedReview in my job would increase my productivity when performing medication reviews	0.217	**0.857**	0.240	0.091	-0.131
Using MedReview would enhance my effectiveness on the job.	0.222	**0.848**	0.216	0.195	0.117
Using MedReview would make it easier to do medication reviews.	0.218	**0.817**	0.265	0.071	0.277
I would find MedReview useful during medication reviews.	0.231	**0.804**	0.298	0.131	0.113
I prefer to be the first one using MedReview.	0.312	0.313	**0.620**	0.148	0.362
Using MedReview gives me an advantage over those who don’t.	0.189	0.420	**0.740**	0.183	0.053
I find it rewarding to use MedReview.	0.305	0.505	**0.600**	0.232	0.260
I could use MedReview if most people around me are using it.	0.146	0.090	**0.634**	0.369	0.020
I could use MedReview if my workplace encourages me to use it.	0.247	0.133	**0.625**	0.434	-0.286
Assuming I have access to MedReview, I intend to use it during medication reviews.	0.213	0.279	**0.774**	0.192	0.138
Given that I have access to MedReview, I predict that I would use it when performing medication reviews.	0.246	0.383	**0.735**	0.155	0.161
I could use MedReview if I have a clear conception of its functionality.	0.160	0.184	0.021	**0.873**	0.022
I could use MedReview if it protects the privacy of its users.	-0.088	0.192	0.260	**0.768**	0.272
I could use MedReview if I feel confident that I can keep it under control.	-0.046	0.173	0.231	**0.784**	0.364
I could use MedReview if I feel confident that the data returned by MedReview is reliable.	0.143	0.030	0.224	**0.819**	-0.087
I could use MedReview if it is meaningful/relevant to my daily tasks.	0.376	0.099	0.298	**0.662**	0.152
I would use MedReview only if it was available for free.	0.204	0.268	0.138	0.397	**0.608**
I could use MedReview if I did not have access to a desktop computer or laptop.	0.318	0.074	0.191	0.482	**0.568**
**Percent of variance (%)**	**20.94**	**19.40**	**14.85**	**14.82**	**6.34**
**α reliability coefficient of each domain**	**0.942**	**0.955**	**0.920**	**0.902**	**0.700**
**Overall scale reliability**	**0.962**				

Note: PE: Perceived ease of use; PIC: Personal initiatives and characteristics; PU: Perceived usefulness; IU: Intention to use; TR: Trust.

The overall reliability (Cronbach’s alpha, α) value of the survey was 0.962, indicating good reliability. All factors were perfect indicators of the dimensions they represent as the reliability of each construct was considered good: perceived ease of use (0.942), perceived usefulness (0.955), intention to use (0.920), trust (0.902), and personal initiatives and characteristics (0.700). Analysis of individual items showed that items were well correlated, as corrected item-total correlation values of individual items were all >0.400 (S3 Table).

### Evaluation of MedReview

#### Qualitative evaluation

‘Expert pharmacists’ of the content validation provided additional feedback regarding MedReview:

- “*Both the guided tool and the simple questionnaire are helpful*”
- “*I found it useful using a tool to review medication*”

There were suggestions to add more resources/references within the MedReview, addition of patient’s national identification number for easier reference, and the potential usefulness of MedReview as a baseline for follow-up reviews.

- “*However*, *it would be good to built in MIMS [Monthly Index of Medical Specialities] or AMH [Australian Medicines Handbook] or BPF [British Pharmaceutical Formulary] reference into the system*, *for immediate checking*. *Especially the potential contraindication or interaction*”
- “*As it is an app*, *it will be good to have the Patient IC [Identity Card] as the common entry instead of name*, *as we may have typo*”
- “*These questions may make more sense if they are a follow up question to if the patient had taken the medication before*”

#### Quantitative evaluation

Descriptive statistics and score distributions of each construct are presented in Table 2, with good variation observed in the data. There was no bunching of scores on either extreme. A slight ceiling effect was observed for ‘trust’ (28.2% of participants selected the highest possible score in the survey) and ‘personal initiatives and characteristics’ (25.5%) constructs, i.e. positively skewed towards the higher end. Floor effects across all constructs were negligible.

**Table 2:** Descriptive statistics and score distributions of the survey constructs for MedReview.

Total score	Number of items	Mean (SD)	Median (IQR)	Possible score range	Actual score range	‘Floor’ effects (worst score) n (%)	‘Ceiling’ effects (best score) n (%)
Perceived ease of use	10	48.72 (10.25)	50.00 (43.00–56.00)	10–70	23–70	29 (2.9)	84 (8.4)
Perceived usefulness	6	29.08 (7.03)	29.00 (25.00–34.75)	6–42	6–42	9 (1.5)	40 (6.7)
Intention to use	7	33.75 (7.61)	35.00 (28.25–39.75)	7–49	13–49	18 (2.6)	68 (9.7)
Trust	5	28.53 (4.86)	29.50 (25.25–32.00)	5–35	11–35	6 (1.2)	141 (28.2)
Personal initiatives and characteristics	2	10.85 (2.35)	11.00 (10.00–13.00)	2–14	2–14	2 (1.0)	51 (25.5)
Overall total score	30	150.93 (26.35)	151.00 (134.75–170.75)	30–210	74–210		

Note: ‘Floor’ and ‘ceiling’ effects % obtained based on the following calculation

[‘Floor’ effect score/(Number of items x number of participants)] x 100.

[‘Ceiling’ effect score/(Number of items x number of participants)] x 100.

The highest mean score (out of 7) achieved for all items was for ‘*I could use MedReview if it is meaningful/relevant to my daily tasks’* (5.78 ± 1.10), followed by ‘*I could use MedReview if I feel confident that the data returned by MedReview is reliable*’ (5.77 ± 1.21), and ‘*I could use MedReview if it protects the privacy of its users’* (5.73 ± 1.20); all three items were from the ‘trust’ construct. The lowest scored item was ‘*I prefer to be the first one using MedReview’* (4.26 ± 1.35) from the ‘intention to use’ construct, and the second lowest was ‘*I would find MedReview to be flexible to interact with’* (4.42 ± 1.29) from ‘perceived ease of use’. These results are summarised in Table 3.

**Table 3:** Descriptive statistics of items in each domain in the survey for MedReview.

Items	Mean (SD)
**Perceived ease of use**	
Learning to operate MedReview would be easy for me.	5.15 (1.31)
I would find it easy to get MedReview to do what I want it to do.	4.72 (1.17)
My interaction with MedReview would be clear and understandable.	4.83 (1.20)
I would find MedReview to be flexible to interact with.	4.42 (1.29)
It would be easy for me to become skilful at using MedReview.	4.94 (1.16)
I would find MedReview easy to use (user-friendly).	4.98 (1.30)
I would find the user interface of MedReview clear and intuitive.	4.76 (1.30)
I am capable of using MedReview.	5.13 (1.34)
I have fun using MedReview.	4.45 (1.26)
I could use MedReview if I am out of home or at my workplace.	5.34 (1.31)
**Perceived usefulness**	
Using MedReview in my job would enable me to accomplish medication reviews more quickly	4.74 (1.32)
Using MedReview would improve my performance in performing a medication review	4.94 (1.22)
Using MedReview in my job would increase my productivity when performing medication reviews	4.82 (1.28)
Using MedReview would enhance my effectiveness on the job	4.77 (1.29)
Using MedReview would make it easier to do medication reviews	4.96 (1.31)
I would find MedReview useful during medication reviews	4.85 (1.35)
**Intention to use**	
I prefer to be the first one using MedReview.	4.26 (1.35)
Using MedReview gives me an advantage over those who don’t.	4.65 (1.27)
I find it rewarding to use MedReview.	4.45 (1.27)
I could use MedReview if most people around me are using it.	4.99 (1.44)
I could use MedReview if my workplace encourages me to use it.	5.35 (1.22)
Assuming I have access to MedReview, I intend to use it during medication reviews.	5.02 (1.38)
Given that I have access to MedReview, I predict that I would use it when performing medication reviews.	5.03 (1.32)
**Trust**	
I could use MedReview if I have a clear conception of its functionality	5.69 (1.02)
I could use MedReview if it protects the privacy of its users	5.73 (1.20)
I could use MedReview if I feel confident that I can keep it under control	5.56 (1.20)
I could use MedReview if I feel confident that the data returned by MedReview is reliable	5.77 (1.21)
I could use MedReview if it is meaningful/relevant to my daily tasks	5.78 (1.10)
**Personal initiatives and characteristics**	
I would use MedReview only if it was available for free	5.54 (1.34)
I could use MedReview if I did not have access to a desktop computer or laptop	5.31 (1.35)

Additional analyses revealed that scores did not vary significantly between gender (see S4 Table). However, there were some significant differences in scores between categories of duration as a practicing community pharmacist. There was a significant difference in mean scores for the ‘perceived usefulness’ construct between those practicing for 0–2 years and 3–4 years (5.464; p = 0.006), and 0–2 years and >9 years (4.078; p = 0.033); the ‘trust’ construct between those practicing for 0–2 years and >9 years (3.307; p = 0.013), and 3–4 years and >9 years (2.947; p = 0.038); and the ‘personal initiatives and characteristics’ construct between those practicing for 0–2 years and >9 years (1.746; p = 0.007). A similar finding was noted for the total overall score with a significant difference between those practicing for 0–2 years and >9 years (16.448; p = 0.024) (see S5 Table).

## Discussion

This study developed, validated and evaluated the use of a CDSS, MedReview, among Malaysian community pharmacists. MedReview was developed to assist healthcare professionals to make pharmacological decisions about continuing, discontinuing or substituting medications for patients. The evaluation component of our study is a user satisfaction survey, and findings indicate that MedReview will be useful for pharmacists conducting medication reviews.

The rationale behind the development of MedReview was based on studies that demonstrated the effectiveness of CDSS during medication reviews. For instance, the Goal-directed Medication Review Electronic Decision Support System (G-MEDSS)^©^ developed in Australia provides clinical decision support to generate patient-specific reports. The reports outline patients’ goals and preferences towards medication use and can be incorporated into Home Medicines Reviews reports [30]. Another tool developed in Australia is the Drug Burden Index^©^, a pharmacological risk assessment measure that quantifies a patient’s exposure to anticholinergic and sedative medications [31]. Developed in the Netherlands, the Systematic Tool to Reduce Inappropriate Prescribing (STRIP) is a drug optimization process to assist physicians with pharmacotherapeutic analysis of medical records during medication reviews [32]. The advantage of MedReview, however, is that it incorporates a scoring scheme and the identification of PIMs for older people alongside pharmacological evaluation; the scores generated are useful for determining improvements (or lack thereof) in medication use over a period of time.

It was essential to evaluate MedReview as a CDSS. The first stage, adaptation of the TAM to evaluate pharmacists’ attitude and intention to use MedReview, showed that our instrument was reliable and internally valid. We used additional constructs proposed by Gao et al. (2011) [21], and our survey instrument had an overall reliability of 0.92, and with subsequent domains achieving reliability of 0.70 or more. Furthermore, our study showed acceptable content validity and fair inter-rater agreement, i.e. all items in our survey instrument were deemed ‘useful’ by two pairs of pharmacists. Factor analysis revealed that the total variance explained by all five domains met the threshold of 70% (76.36%), suggesting that it was meaningful to retain five domains.

The TAM is widely used and is the most accepted of various models described in the literature [20–22]. In their study on measurement of the adoption of mobile services, Gao et al. (2011) introduced some antecedents to the TAM which included new constructs such as trust, personal characteristics and initiatives, context and intention to use [21]. Internal consistency/reliability in their study was above 0.7 for all domains, indicating high reliability of the scale. Item-to-item correlation and convergent and discriminant validity provided evidence for construct validity. More recently, Weng et al. (2018) conducted a TAM-based study of attitudes towards intention to use multimedia among school teachers [20]. They adapted the TAM to include constructs such as attitude toward using and intention to use, in addition to perceived ease of use and usefulness. The internal consistency of all domains was above 0.70. Hussain et al. (2016) also adapted the TAM to study users’ intention of use and their acceptance of an interactive mobile map using these constructs: perceived ease of use, usefulness and enjoyment [22]. The internal consistency and composite reliability of their questionnaire was above 0.70, with good convergent (factor loadings greater than 0.50) and discriminant (no high cross-loadings of items) validity.

Using a valid and reliable instrument to evaluate MedReview provides confidence in our findings about community pharmacists’ perceptions. The majority of pharmacists had a positive attitude towards MedReview. Of the various aspects of the adapted TAM in this study, pharmacists scored highest in terms of trusting MedReview and having the initiative to use it, followed by intention to use. Cultivating user’s trust is known to be time-consuming and it is challenging to gain, but easy to lose [21]. Therefore, the highest score—which was achieved for trust—is promising because a user’s perception of the security and privacy aspects, as well as the integrity of the tool, remain fundamental antecedents of trust. Pharmacists in our study felt that they would use MedReview if it was relevant to their daily tasks, if the data returned was reliable, and if it protected the privacy of users. MedReview anonymizes patient information and does not disclose information to third parties, except a summary of the review emailed to the user. The initiative and intention to use a tool such as MedReview could be due to various reasons [3]. First, MedReview is capable of ensuring a systematic and structured review due to the guided framework embedded within it, and is stepwise in nature. It is also time-efficient and can consolidate individuals’ information, thereby negating unnecessary documentation, speeding up the process. MedReview could be cost-efficient because a one-off investment in the system may offset long-term costs associated with current processes. MedReview may also encourage process integration at RACFs and other healthcare settings, as the integration of information would ensure that GPs and personnel at RACFs have easy access to an individual’s records promoting continuity of care. Data storage for quality improvement is possible as MedReview has the potential to record outcomes from reviews; data could also be useful for confirming when previous reviews were conducted. Data has the potential to be used by policy-makers and for audit, education and research purposes. Medication optimization using MedReview is possible even when face-to-face interactions are restricted [3].

However, pharmacists were less likely to feel that MedReview was ‘easy to use’ and ‘useful’. This may be because they used MedReview for the first time in this study, and it has been reported that first time-users may have some difficulty navigating and foreseeing potential usefulness [33]. Perceived ease of use is described by Davis et al. (1989) as ‘*the extent to which the user believes using a particular system will be free of effort*’ [19], i.e. using a specific technology like MedReview should be free of mental and physical exhaustion [22]. Perceived usefulness on the other hand, is described as ‘*the extent to which the user believes using a particular system would enhance their job performance*’ [19]. In the current study, perceived usefulness explains pharmacists’ recognition that MedReview would enhance their performance if they were to conduct medication reviews. Possible reasons for lower scores in these domains could be that pharmacists could foresee a potential disruption in workflow when doing a medication review, which could lead to loss of productivity, and negative emotions about learning and adopting a new system [34].

Gender did not have a significant impact on the use of MedReview, which is at odds with a study by He and Freeman (2009) that revealed men had a more encouraging attitude and self-efficacy toward the use of technology [35]. In our study this might have been due to a smaller sample size and spread of the data. Adding to that, community pharmacists with fewer years of experience generally found MedReview more useful and trustworthy and had the initiative to use it compared to pharmacists with more years of experience (longer than nine years). This could be due to the age factor, where older pharmacists may have perceived barriers about using technology, such as complexity, feeling of inadequacy, and scepticism about using technology in general [36]. The free text written (qualitative) feedback from expert pharmacists during the validation stages was generally positive, as they indicated that MedReview was useful and helpful. Suggestions included design improvements, some of which include incorporating additional references.

This study had some limitations. Selection bias may have been introduced through convenience rather than random sampling, reducing generalizability of the results; however, the inclusion of pharmacists from various regions of Malaysia may have compensated to some extent. Reporting bias may have been introduced by the self-report nature of our study. The ‘online’ nature of the study may have been time-consuming, as pharmacists had to familiarize themselves with MedReview, the study instructions and cases. There were also strengths, particularly the use of technology to conduct medication reviews, which provides a novel platform to streamline the process. The scoring method in MedReview could be useful in longitudinal studies investigating the effectiveness of medication review interventions. The use of the TAM as a theoretical framework to evaluate use of MedReview provided reliable insight pertaining to its use and validation in our study allows for replication in future larger scale studies.

### Implications of MedReview in practice and research

Medication reviews are not a formalized and remunerated service provided by community pharmacists in Malaysia; however, MedReview provides a potential platform for conducting reviews. It may also encourage more community pharmacists to conduct reviews for patients who may be vulnerable to adverse medication effects and poor medication adherence, particularly older people.

Health care reform in Malaysia, and particularly the concept of “1 Care”, is the proposed restructuring of the healthcare system with a main focus on primary healthcare [37]. Of the main concerns underpinning this reform, management of chronic diseases and maintaining patient expectations and safety are both relevant to quality medication reviews performed by community pharmacists [12]. Pharmacist-led medication reviews are established and remunerated services in many developed countries such as Australia, the United Kingdom and the United States of America [38–41], and MedReview could be used to initiate this service in community pharmacies in Malaysia and elsewhere. However, its use need not be restricted to this setting as future studies can assess the use of MedReview in government and private hospitals and health clinics, as well as in RACFs.

## Conclusions

This study developed, validated and evaluated MedReview for healthcare professionals conducting medication reviews, particularly for older people. The validation and evaluation of MedReview among community pharmacists indicated a positive attitude towards this CDSS tool for medication reviews in a community pharmacy setting. Pharmacists found that MedReview is trustworthy and they had the intention to use it when conducting medication reviews. Findings also suggest that adaptation of the TAM to assess the intention and attitude of pharmacists in using MedReview, was reliable and internally valid.

## Supporting information

S1 Fig Scree plot for the factor analysis of 5 domains. (DOCX)

S1 Table Demographic characteristics of study participants. (DOCX)

S2 Table Inter-rater agreement of two pairs of pharmacists for the survey items used to assess the MedReview tool (content validation). (DOCX)

S3 Table Item and reliability analysis. (DOCX)

S4 Table Mean comparisons of total scores for each domain for gender. (DOCX)

S5 Table Mean comparisons of total scores for each domain for duration of being community pharmacists. (DOCX)
