# Supplementary figures and images for: Development, validation and evaluation of an online medication review tool (MedReview)
Source: PLoS One. 2022 Jun 3;17(6):e0269322. doi: 10.1371/journal.pone.0269322 (PMC9165870; doi:10.1371/journal.pone.0269322)

**S1 Fig. Scree plot for the factor analysis of 5 domains.**


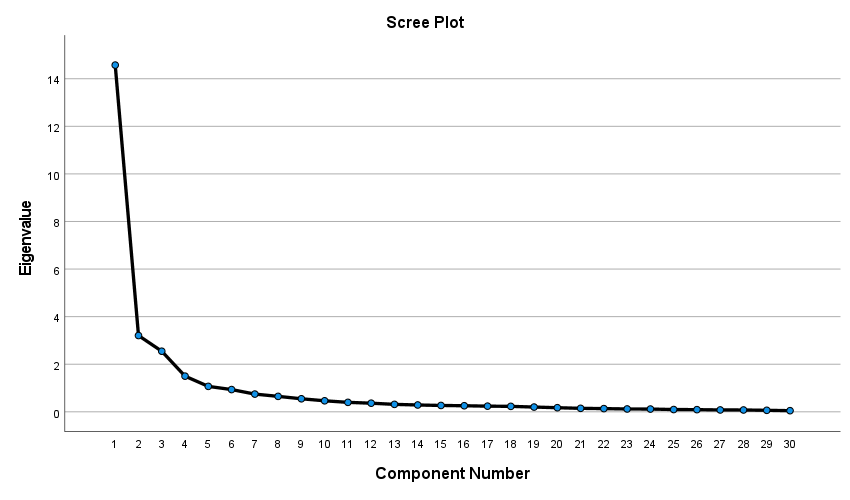

Supplement: S1 Fig — (DOCX) [file pone.0269322.s001.docx]
