# Supplementary material for: Development, validation and evaluation of an online medication review tool (MedReview)
Source: PLoS One. 2022 Jun 3;17(6):e0269322. doi: 10.1371/journal.pone.0269322 (PMC9165870; doi:10.1371/journal.pone.0269322)
Supplement: S1 Table — (DOCX) [file pone.0269322.s002.docx]

**S1 Table. Demographic characteristics of study participants.**

| **Participant characteristics** | **n (%)** |
| --- | --- |
| n | 100 |
| **Age categories** | |
| <25 years | 7 (7) |
| 26-35 years | 68 (68) |
| 36-45 years | 19 (19) |
| >45 years | 6 (6) |
| **Geographical location of study participants** |  |
| Selangor | 27 (27) |
| Johor | 19 (19) |
| Kuala Lumpur | 17 (17) |
| Negeri Sembilan | 12 (12) |
| Sarawak | 5 (5) |
| Penang | 5 (5) |
| Melaka | 4 (4) |
| Terengganu | 3 (3) |
| Perak | 3 (3) |
| Pahang | 2 (2) |
| Kelantan | 1 (1) |
| Kedah | 1 (1) |
| Sabah | 1 (1) |
| **Gender** | |
| Male | 35 (35) |
| Female | 65 (65) |
| **Academic qualifications** | |
| Bachelor of Pharmacy | 88 (88) |
| Masters | 12 (12) |
| Doctor of Philosophy (PhD) | 0 |
| Postdoctoral degree | 0 |
| **Duration of being Fully Registered Pharmacists** | |
| <5 years | 46 (46) |
| 6-10 years | 27 (27) |
| 11-20 years | 20 (20) |
| >20 years | 7 (7) |
| **Duration of being community pharmacists** | |
| 0-2 years | 29 (29) |
| 3-4 years | 22 (22) |
| 5-9 years | 24 (24) |
| >9 years | 25 (25) |
