# Supplementary material for: Development, validation and evaluation of an online medication review tool (MedReview)
Source: PLoS One. 2022 Jun 3;17(6):e0269322. doi: 10.1371/journal.pone.0269322 (PMC9165870; doi:10.1371/journal.pone.0269322)
Supplement: S2 Table — (DOCX) [file pone.0269322.s003.docx]

**S2 Table. Inter-rater agreement of two pairs of pharmacists for the survey items used to assess the MedReview tool (content validation).**

| **Items in each survey construct** | **First rater pair** | | | | | **Second rater pair** | | | | | **Overall agreement of usefulness** |
| --- | --- | --- | --- | --- | --- | --- | --- | --- | --- | --- | --- |
|  | **A** | **B** | **C** | **D** | **Agreement of usefulness** | **A** | **B** | **C** | **D** | **Agreement of usefulness** |  |
| **Perceived ease of use** | | | | | | | | | | | |
| Learning to operate MedReview would be easy for me. | 0 | 0 | 1 | 0 | 0 | 1 | 0 | 0 | 0 | 100 | 50 |
| I would find it easy to get MedReview to do what I want it to do. | 1 | 0 | 0 | 0 | 100 | 1 | 0 | 0 | 0 | 100 | 100 |
| My interaction with MedReview would be clear and understandable. | 1 | 0 | 0 | 0 | 100 | 1 | 0 | 0 | 0 | 100 | 100 |
| I would find MedReview to be flexible to interact with. | 1 | 0 | 0 | 0 | 100 | 1 | 0 | 0 | 0 | 100 | 100 |
| It would be easy for me to become skillful at using MedReview. | 1 | 0 | 0 | 0 | 100 | 1 | 0 | 0 | 0 | 100 | 100 |
| I would find MedReview easy to use (user-friendly). | 1 | 0 | 0 | 0 | 100 | 1 | 0 | 0 | 0 | 100 | 100 |
| I would find the user interface of MedReview clear and intuitive. | 1 | 0 | 0 | 0 | 100 | 1 | 0 | 0 | 0 | 100 | 100 |
| I am capable of using MedReview. | 0 | 0 | 1 | 0 | 0 | 1 | 0 | 0 | 0 | 100 | 50 |
| I have fun using MedReview. | 0 | 0 | 1 | 0 | 0 | 1 | 0 | 0 | 0 | 100 | 50 |
| I could use MedReview if I am out of home or at my workplace. | 1 | 0 | 0 | 0 | 100 | 1 | 0 | 0 | 0 | 100 | 100 |
| **Perceived usefulness** | | | | | | | | | | | |
| Using MedReview in my job would enable me to accomplish medication reviews more quickly | 1 | 0 | 0 | 0 | 100 | 1 | 0 | 0 | 0 | 100 | 100 |
| Using MedReview would improve my performance in performing a medication review | 1 | 0 | 0 | 0 | 100 | 1 | 0 | 0 | 0 | 100 | 100 |
| Using MedReview in my job would increase my productivity when performing medication reviews | 1 | 0 | 0 | 0 | 100 | 1 | 0 | 0 | 0 | 100 | 100 |
| Using MedReview would enhance my effectiveness on the job | 0 | 0 | 1 | 0 | 0 | 1 | 0 | 0 | 0 | 100 | 50 |
| Using MedReview would make it easier to do medication reviews | 0 | 0 | 1 | 0 | 0 | 1 | 0 | 0 | 0 | 100 | 50 |
| I would find MedReview useful during medication reviews | 1 | 0 | 0 | 0 | 100 | 1 | 0 | 0 | 0 | 100 | 100 |
| **Intention to use** | | | | | | | | | | | |
| I prefer to be the first one using MedReview. | 0 | 0 | 1 | 0 | 0 | 1 | 0 | 0 | 0 | 100 | 50 |
| Using MedReview gives me an advantage over those who don't. | 0 | 1 | 0 | 0 | 0 | 1 | 0 | 0 | 0 | 100 | 50 |
| I find it rewarding to use MedReview. | 0 | 0 | 1 | 0 | 0 | 1 | 0 | 0 | 0 | 100 | 50 |
| I could use MedReview if most people around me are using it. | 0 | 0 | 0 | 1 | 100 | 1 | 0 | 0 | 0 | 100 | 100 |
| I could use MedReview if my workplace encourages me to use it. | 1 | 0 | 0 | 0 | 100 | 1 | 0 | 0 | 0 | 100 | 100 |
| Assuming I have access to MedReview, I intend to use it during medication reviews. | 1 | 0 | 0 | 0 | 100 | 1 | 0 | 0 | 0 | 100 | 100 |
| Given that I have access to MedReview, I predict that I would use it when performing medication reviews. | 0 | 0 | 1 | 0 | 0 | 1 | 0 | 0 | 0 | 100 | 50 |
| **Trust** | | | | | | | | | | | |
| I could use MedReview if I have a clear conception of its functionality | 1 | 0 | 0 | 0 | 100 | 1 | 0 | 0 | 0 | 100 | 100 |
| I could use MedReview if it protects the privacy of its users | 1 | 0 | 0 | 0 | 100 | 1 | 0 | 0 | 0 | 100 | 100 |
| I could use MedReview if I feel confident that I can keep it under control | 1 | 0 | 0 | 0 | 100 | 1 | 0 | 0 | 0 | 100 | 100 |
| I could use MedReview if I feel confident that the data returned by MedReview is reliable | 1 | 0 | 0 | 0 | 100 | 1 | 0 | 0 | 0 | 100 | 100 |
| I could use MedReview if it is meaningful/relevant to my daily tasks | 1 | 0 | 0 | 0 | 100 | 1 | 0 | 0 | 0 | 100 | 100 |
| **Personal initiatives and characteristics** | | | | | | | | | | | |
| I would use MedReview only if it was available for free | 1 | 0 | 0 | 0 | 100 | 1 | 0 | 0 | 0 | 100 | 100 |
| I could use MedReview if I did not have access to a desktop computer or laptop | 1 | 0 | 0 | 0 | 100 | 1 | 0 | 0 | 0 | 100 | 100 |

A= Useful according to both pharmacists; B= Useful according to Pharmacist 1, but not to Pharmacist 2; C= Useful according to Pharmacist 2, but not to Pharmacist 1; D= Not useful according to both pharmacists

Agreement= (A+D)/(A+B+C+D) x 100

Overall agreement of usefulness= (Agreement of usefulness of first rater pair + agreement of usefulness of second rater pair)/2
