# Supplementary material for: Development, validation and evaluation of an online medication review tool (MedReview)
Source: PLoS One. 2022 Jun 3;17(6):e0269322. doi: 10.1371/journal.pone.0269322 (PMC9165870; doi:10.1371/journal.pone.0269322)
Supplement: S3 Table — (DOCX) [file pone.0269322.s004.docx]

**S3 Table. Item and reliability analysis.**

| **Items** | | **Scale mean if item deleted** | **Corrected item-total correlation** | | **Cronbach’s alpha if item deleted** | |
| --- | --- | --- | --- | --- | --- | --- |
| **Perceived ease of use** | | |  | |  | |
| Learning to operate MedReview would be easy for me. | | 43.57 | 0.737 | | 0.937 | |
| I would find it easy to get MedReview to do what I want it to do. | | 44.00 | 0.816 | | 0.934 | |
| My interaction with MedReview would be clear and understandable. | | 43.89 | 0.815 | | 0.933 | |
| I would find MedReview to be flexible to interact with. | | 44.30 | 0.693 | | 0.939 | |
| It would be easy for me to become skilful at using MedReview. | | 43.78 | 0.819 | | 0.933 | |
| I would find MedReview easy to use (user-friendly). | | 43.74 | 0.837 | | 0.932 | |
| I would find the user interface of MedReview clear and intuitive. | | 43.96 | 0.810 | | 0.933 | |
| I am capable of using MedReview. | | 43.59 | 0.723 | | 0.938 | |
| I have fun using MedReview. | | 44.27 | 0.760 | | 0.936 | |
| I could use MedReview if I am out of home or at my workplace. | | 43.38 | 0.627 | | 0.942 | |
| **Perceived usefulness** |  | | |  | |  |
| Using MedReview in my job would enable me to accomplish medication reviews more quickly | | 24.34 | 0.852 | | 0.948 | |
| Using MedReview would improve my performance in performing a medication review | | 24.14 | 0.818 | | 0.951 | |
| Using MedReview in my job would increase my productivity when performing medication reviews | | 24.26 | 0.852 | | 0.948 | |
| Using MedReview would enhance my effectiveness on the job | | 24.31 | 0.893 | | 0.943 | |
| Using MedReview would make it easier to do medication reviews | | 24.12 | 0.885 | | 0.944 | |
| I would find MedReview useful during medication reviews | | 24.23 | 0.862 | | 0.947 | |
| **Intention to use** |  | | |  | |  |
| I prefer to be the first one using MedReview. | | 29.49 | 0.732 | | 0.910 | |
| Using MedReview gives me an advantage over those who don't. | | 29.10 | 0.829 | | 0.900 | |
| I find it rewarding to use MedReview. | | 29.30 | 0.827 | | 0.900 | |
| I could use MedReview if most people around me are using it. | | 28.76 | 0.625 | | 0.922 | |
| I could use MedReview if my workplace encourages me to use it. | | 28.40 | 0.648 | | 0.918 | |
| Assuming I have access to MedReview, I intend to use it during medication reviews. | | 28.73 | 0.805 | | 0.902 | |
| Given that I have access to MedReview, I predict that I would use it when performing medication reviews. | | 28.72 | 0.817 | | 0.901 | |
| **Trust** |  | | |  | |  |
| I could use MedReview if I have a clear conception of its functionality | | 22.84 | 0.783 | | 0.876 | |
| I could use MedReview if it protects the privacy of its users | | 22.80 | 0.768 | | 0.877 | |
| I could use MedReview if I feel confident that I can keep it under control | | 22.97 | 0.809 | | 0.868 | |
| I could use MedReview if I feel confident that the data returned by MedReview is reliable | | 22.76 | 0.765 | | 0.878 | |
| I could use MedReview if it is meaningful/relevant to my daily tasks | | 22.75 | 0.661 | | 0.899 | |
| **Personal initiatives and characteristics** |  | | |  | |  |
| I would use MedReview only if it was available for free | | 5.31 | 0.532 | | - | |
| I could use MedReview if I did not have access to a desktop computer or laptop | | 5.54 | 0.532 | | - | |
