# Supplementary material for: Development, validation and evaluation of an online medication review tool (MedReview)
Source: PLoS One. 2022 Jun 3;17(6):e0269322. doi: 10.1371/journal.pone.0269322 (PMC9165870; doi:10.1371/journal.pone.0269322)
Supplement: S4 Table — (DOCX) [file pone.0269322.s005.docx]

**S4 Table. Mean comparisons of total scores for each domain for gender.**

| **Total score** | **Gender** | **n** | **Mean (SD)** | **p-value** |
| --- | --- | --- | --- | --- |
| Perceived ease of use | Male | 35 | 46.23 (10.33) | 0.074 |
|  | Female | 65 | 50.06 (10.04) |  |
| Perceived usefulness | Male | 35 | 28.54 (6.92) | 0.578 |
|  | Female | 65 | 29.37 (7.13) |  |
| Intention to use | Male | 35 | 33.26 (7.29) | 0.637 |
|  | Female | 65 | 34.02 (7.82) |  |
| Trust | Male | 35 | 28.51 (5.59) | 0.981 |
|  | Female | 65 | 28.54 (4.46) |  |
| Personal initiatives and characteristics | Male | 35 | 10.40 (2.40) | 0.162 |
|  | Female | 65 | 11.09 (2.31) |  |
| Total overall score | Male | 35 | 146.94 (26.38) | 0.269 |
|  | Female | 65 | 153.08 (26.29) |  |

Note: The actual score range for each domain are as follows:

Perceived ease of use: 23-70; perceived usefulness: 6-42; intention to use: 13-49; trust: 11-35; personal initiatives and characteristics: 2-14; total overall score: 74-210
