# Supplementary material for: Development, validation and evaluation of an online medication review tool (MedReview)
Source: PLoS One. 2022 Jun 3;17(6):e0269322. doi: 10.1371/journal.pone.0269322 (PMC9165870; doi:10.1371/journal.pone.0269322)
Supplement: S5 Table — (DOCX) [file pone.0269322.s006.docx]

**S5 Table. Mean comparisons of total scores for each domain for duration of being community pharmacists.**

| **Domains** | **Duration of being community pharmacists**  **(I)** | **Duration of being community pharmacists**  **(J)** | **Mean difference**  **(I-J)** | **p-value** |
| --- | --- | --- | --- | --- |
| Perceived ease of use | 0-2 years | 3-4 years | 1.868 | 0.521 |
|  |  | 5-9 years | 0.774 | 0.783 |
|  |  | >9 years | 4.539 | 0.112 |
|  | 3-4 years | 5-9 years | -1.095 | 0.716 |
|  |  | >9 years | 2.670 | 0.380 |
|  | 5-9 years | >9 years | 3.765 | 0.202 |
| Perceived usefulness | 0-2 years | 3-4 years | **5.464** | 0.006 |
|  |  | 5-9 years | 2.268 | 0.226 |
|  |  | >9 years | **4.078** | 0.033 |
|  | 3-4 years | 5-9 years | -3.196 | 0.112 |
|  |  | >9 years | -1.386 | 0.493 |
|  | 5-9 years | >9 years | 1.810 | 0.355 |
| Intention to use | 0-2 years | 3-4 years | 0.998 | 0.646 |
|  |  | 5-9 years | 0.902 | 0.667 |
|  |  | >9 years | 2.779 | 0.192 |
|  | 3-4 years | 5-9 years | -0.096 | 0.966 |
|  |  | >9 years | 1.780 | 0.433 |
|  | 5-9 years | >9 years | 1.877 | 0.393 |
| Trust | 0-2 years | 3-4 years | 0.361 | 0.789 |
|  |  | 5-9 years | 1.284 | 0.325 |
|  |  | >9 years | **3.307** | 0.013 |
|  | 3-4 years | 5-9 years | 0.924 | 0.508 |
|  |  | >9 years | **2.947** | 0.038 |
|  | 5-9 years | >9 years | 2.023 | 0.140 |
| Personal initiatives and characteristics | 0-2 years | 3-4 years | 0.757 | 0.248 |
|  |  | 5-9 years | 0.741 | 0.241 |
|  |  | >9 years | **1.746** | 0.007 |
|  | 3-4 years | 5-9 years | -0.016 | 0.981 |
|  |  | >9 years | 0.989 | 0.149 |
|  | 5-9 years | >9 years | 1.005 | 0.130 |
| Total overall score | 0-2 years | 3-4 years | 9.448 | 0.202 |
|  |  | 5-9 years | 5.968 | 0.403 |
|  |  | >9 years | **16.448** | 0.024 |
|  | 3-4 years | 5-9 years | -3.480 | 0.649 |
|  |  | >9 years | 7.000 | 0.365 |
|  | 5-9 years | >9 years | 10.480 | 0.162 |

Note: Values in bold indicate statistical significance at the 0.05 level

Scale range for each domain are 1 (worst score) to 7 (best score)
